# Le rôle du médecin anesthésiste-réanimateur dans la prise en charge de la femme enceinte porteuse de la maladie de Von Willebrand

**DOI:** 10.11604/pamj.2015.22.335.7997

**Published:** 2015-12-04

**Authors:** Hanane Baouahi, Yassine Zerqouni, Mouhcine Doumiri, Nezha Oudghiri, Anas Tazi Saoud

**Affiliations:** 1 Service d'Anesthésie–Réanimation, Hôpital Maternité Souissi, Centre Hospitalier Universitaire, Université Mohamed V Rabat, Maroc

**Keywords:** Maladie de Von Willebrand, risque hémorragique, péripartum, Von Willebrand disease, hemorrhagic risk, peripartum

## Abstract

La maladie de Von Willebrand (VWD) est la maladie hémorragique constitutionnelle de l'hémostase la plus fréquente. Elle est liée à un déficit, soit quantitatif, soit qualitatif en facteur willebrand (VWF). Elle se caractérise par son extrême hétérogénéité sur les plans clinique, phénotypique et génotypique. La grossesse et surtout le péri-partum représente une période à risque hémorragique pour ces femmes. Nous rapportons le cas d'une parturiente présentant une maladie de Von Willebrand de type 1 documentée, la difficulté du choix du mode d'accouchement et de la technique anesthésique a été revue.

## Introduction

La maladie de Von Willebrand est un trouble héréditaire de la coagulation du à une carence ou un dysfonctionnement du facteur de von Willebrand (VWF), Une protéine plasmatique qui médie l'adhérence initiale des plaquettes à des sites de lésion vasculaire et se lie également et stabilise le facteur de coagulation sanguine VIII (FVIII) dans la circulation. Les défauts de VWF peuvent causer un saignement en altérant l'adhésion des plaquettes ou en réduisant la concentration de FVIII [1]. Sa prévalence est estimée entre 0.6% et 1.3% avec une prédominance féminine.

## Patient et observation

Mme H.E; 23 ans, primipare; 41SA + 1j (DDR = 28/02/2013) ayant comme antécédents: Maladie de Von Willebrand de type 1 diagnostiquée à l’âge de 10 ans après hémorragie ORL suite à une amygdalectomie et des Ménorragies importantes nécessitant une fois la transfusion par des CGR et des PFC.

**Examen à l'admission:** patiente consciente; Conjonctives normocolorées, FC = 80bpm; TA =120/60mmHg; FR = 16cpm auscultation cardiopulmonaire normale, Pas syndrome hémorragique cutanéo-muqueux, examen ostéo-articulaire est sans partucularité. **Examen obstétrical:** MAF présents, pas de CU,BCF = +,HU= 29cm, TV= Col long fermé postérieur, Présentation céphalique mobile, Poche des eaux intacte Rcf: normal Echographie obstétricale: Grossesse monofoetale évolutive en présentation céphalique, BIP = 94mm; LF = 69mm, Placenta antéro-fundique, Liquide amniotique de quantité normale, **Biologie:** plaquettes= 234 G/L,Temps de saignement allongé (méthode d'Ivy)= 13min,TCA= 30sec (1T),TP= 76.6%, INR= 1.26, Fibrinogène= 3.40 g, Facteur VIIIc (Chronométrie): 9%- N= 50-150%, Facteur Von Willebrand (Immunoturbidemétrie): 21% - N= 50-160%, Facteur Von Willebrand (Agrégométrie) / Activité cofacteur de la ristocétine du FvW: < 10% -N= 50-150%.

## Discussion

Le facteur de Von Willebrand est une protéine multimérique formée d'une série de 15 à 20 multimères de poids moléculaires variables Synthétisée par les cellules endothéliales et les mégacaryocytes ayant une demie-vie de 12 heures et ayant deux grandes fonctions, l'adhésion des plaquettes à la paroi vasculaire lésée et le transport et la protection du facteur VII. Il existe trois types de MvW [1]: le type 1 est la forme la plus fréquente représentant 60 à 80% des cas. Elle est caractérisée par un déficit quantitatif partiel en FvW; le type 2 correspond à une synthèse quantitativement normale ou subnormale de FvW qui est qualitativement anormal. On décrit quatre variantes: le type 2A caractérisé par un défaut de liaison de FvW aux plaquettes lié à l'absence des multimères de haut poids moléculaire et de poids moléculaire intermédiaire; le type 2B caractérisé par une augmentation anormale de l'affinité du FvW pour la GPIb/IX des plaquettes même sans lésion vasculaire, ce qui provoque thrombopénie; le type 2M caractérisé par une diminution de l'adhésion des plaquettes dépendante du VWF sans déficit sélectif des multimères de haut poids moléculaire; le type 2N caractérisé par un défaut de liaison du FvW au Facteur VIII; le type 3 est la forme la plus sévère, et la moins fréquente (moins de 5%), caractérisé par des taux extrêmement bas ou indétectables de FvW et de son activité. **L’évaluation** d'une personne pour une éventuelle maladie de Von Willebrand ou d'autres troubles de la coagulation **peut être engagée** en raison de **diverses indications cliniques** [1]. Ces indications sont: une personne asymptomatique qui va subir une chirurgie ou procédure interventionnelle invasive; les personnes qui se présentent avec des: antécédents de saignements abondants; antécédents familiaux de troubles de la coagulation; symptômes hémorragiques; études de laboratoire anormales; une combinaison de ces éléments. Les personnes qui se présentent avec un diagnostic préalable de maladie de Von Willebrand, mais qui ne possèdent pas les pièces justificatives de laboratoire. Une première évaluation de l'hémostase comprend généralement: Un nombre de plaquettes et une numération sanguine, TCA, le temps de prothrombine et éventuellement, soit un taux de fibrinogène ou un temps de thrombine [1]. Ce test **ne peut ni affirmer ni infirmer la VWD**, mais il **peut suggérer** que la carence en facteur de coagulation ou thrombopénie pourrait être la cause potentielle d'une hémorragie clinique.

Tests spécialisés: l’étude de l'agrégation plaquettaire en présence de ristocétine (RIPA); la distribution des multimères du FvW déterminée par électrophorèse du plasma le dosage du FvW intraplaquettaire Etude de la liaison du FvW aux plaquettes, au collagène et au fac VIII. Analyse de l'ADN permettant la détection de défauts génétiques. Durant la grossesse, les taux de FvW et de facteur VIII augmentent à partir de la 16e SA jusqu’à atteindre trois à quatre fois la valeur observée avant la grossesse. Cette élévation physiologique des facteurs de la coagulation peut être suffisante pour une bonne hémostase durant la grossesse et l'accouchement chez les femmes enceintes porteuses d'affections hémorragiques congénitales. Cependant, ces facteurs chutent rapidement après l'accouchement. Ainsi, les désordres hémorragiques sont rares durant la grossesse et surviennent surtout pendant l'accouchement et en postpartum [2]. Dans notre cas, Facteur VIII activité: 120% à 38SA et RIPA: 80%. Le but du traitement est de prévenir ou traiter l'hémorragie. Deux possibilités thérapeutiques majeures disponibles, la desmopressine (1-déamino-8-D-arginine vasopressine) et les concentrés plasmatiques de facteur Willebrand, plus ou moins riches en facteur VIII. Le choix dépend de: Type et de la gravité de la maladie, la réponse à la dDAVP et la situation clinique c'est-à-dire de l'importance du saignement et du temps nécessaire pour corriger l'anomalie de l'hémostase. Tout en respectant les contre-indications: aspirine, AINS… La desmopressine est un analogue synthétique d'arginine-vasopressine. Elle provoque la libération, par les cellules endothéliales, de VWF endogène augmentant ainsi son taux dans la circulation sanguine mais aussi celui du facteur VIII ce qui permet une correction transitoire (quelques heures) du déficit. Des dosages en VWF et en FVIII sont réalisés généralement avant l'injection de desmopressine puis une heure, voire deux heures, quatre heures ou parfois huit heures après. L'effet de la desmopressine est cependant transitoire (6 à 8 heures) et les doses doivent donc être répétées toutes les 12 à 24 heures. La posologie de la desmopressine est de 0,3kg/mcg en intraveineux dans 30-50 ml de solution saline normale pendant 30 minutes, avec des pics de FVIII et VWF 30-90 min après la perfusion [3]. L'administration nasale de desmopressine acétate à haute dose est souvent efficace pour un saignement mineur, mais la voie IV est la voie préférée pour la prophylaxie de l'hémorragie chirurgicale et pour le traitement de l'hémorragie importante; l'absorption nasale est variable et tous les patients atteints de maladie de von Willebrand et qui sont sensibles à desmopressine devrait subir un test après instillation nasale pour mesurer reponse FVIII et VWF avant de l'utiliser. Lorsqu'il est utilisé pour épistaxis, l'administration nasale est délivrée dans la narine qui ne saigne pas [3]. La desmopressine peut également être administrée par voie sous-cutanée (sc). La posologie efficace est identique à la dose IV, mais la préparation SC n'est pas disponible. La réponse à la desmopressine diminue avec des doses répétées, probablement en raison de l’épuisement du compartiment de stockage de VWF [3]. En plus de tachyphylaxie, hyponatrémie peut compliquer l'utilisation répétée de la desmopressine. La restriction hydrique et le suivi de sodium sérique sont recommandés [3]. L'utilisation de DDAVP pendant la grossesse est controversée en raison du risque théorique de retard de croissance intra-utérin, la contraction utérine et d'accouchement prématuré Une revue systématique et une série de cas rapportent une bonne innocuité et une efficacité de DDAVP [4]. DDAVP administrée aux femmes enceintes dans leur premier et deuxième trimestres avant les procédures invasives (c. prélèvement de villosités choriales, amniocentèse, cerclage du col utérin, résiliation) et pour le traitement d'une hémorragie du premier trimestre n'a pas provoqué de complications néonatales, et des effets secondaires bénins maternels ont été rapportés. Lorsque administeré en peripartum aux femmes atteintes de troubles hémorragiques, DDAVP empêche l'hémorragie du post-partum dans 167 des 172 livraisons (97%). Parmi les cinq grossesses dans lesquelles événements hémorragiques indésirables ont été rapportés, il y'avait quatre cas d'hémorragie du post-partum et un cas d'hématome sous-cutané. Les troubles de la coagulation sous-jacents des femmes qui ont développé des complications hémorragiques malgré un traitement comprenaient deux cas de syndrome Hermansky - Pudlak et un seul cas chacun de trouble plaquettaire, EDS et maladie de Von Willebrand. D'autres effets indésirables associés à l'utilisation de DDAVP pendant la grossesse comprenaient un cas d'accouchement prématuré dans le neuvième mois et un cas de crise convulsive résultant de l'hyponatrémie [4]. Aucune complication néonatale ou des signes de retard de croissance intra-utérin n'ont été rapportés. Il n'ya pas eu des rapports d'effets tératogènes chez les fœtus exposés à la DDAVP dans le premier trimestre de la grossesse, et les modèles in vitro montrent que la DDAVP ne traverse pas la barrière placentaire. DDAVP est libérée dans le lait maternel en très petites quantités, absorption orale négligeable, ce qui suggère que la consommation maternelle pendant l'allaitement est sûre. Néanmoins, le fabricant déconseille l'utilisation du DDAVP par les mères qui allaitent. Les concentrés plasmatiques de facteur Willebrand sont des traitements de substitution administrés par voie intra veineuse sous forme de concentrés de facteurs Willebrand plus ou moins riches en facteur VIII, ils représentent la seule possibilité thérapeutique pour les patients qui ne peuvent bénéficier de dDAVP. Ces concentrés sont obtenus par purification de plasma de donneurs sains. Ils sont traités par solvant détergent ce qui élimine le risque de transmission de virus à enveloppe lipidique (Virus de l'immunodéficience humaine (VIH), virus des hépatites B et C (VHB, VHC)) mais laisse persister un risque de transmission de virus nus (parvovirus, virus de l'hépatite A…). Il est donc recommandé de vacciner ces patients contre l'hépatite A, mais aussi contre l'hépatite B puisqu'ils sont exposés à recevoir des produits sanguins labiles. Ces concentrés sont efficaces dans tous les types de VWD mais du fait du risque résiduel de transmission virale et de leur coût très élevé, ils doivent être réservés aux patients qui ne peuvent bénéficier d'un traitement par la dDAVP en raison d'une inefficacité, de la situation clinique (hémorragie majeure, chirurgie importante…), d'une tachyphylaxie ou d'une contre indication. Le VWF et ee FVIII augmentent considérablement pendant la grossesse chez les femmes normales et la plus forte augmentation est évidente au cours du troisième trimestre, avec des niveaux excessivement > 100 U / dL au moment de l'accouchement. Comme les femmes enceintes atteintes de maladie de von Willebrand sont à risque accru d'hémorragie du post-partum si non traitées, des options thérapeutiques doivent être déjà prévues pour le début de la grossesse. Une prise en charge invasive de l'accouchement avec ventouse, forceps, etc doit être évitée pendant le risque de saignement pour le nouveau-né potentiellement affecté. Une grande hétérogénéité de phénotypes et des mécanismes physiopathologiques sous-jacents sont associés à ce trouble, une évaluation rapide et soigneuse des femmes enceintes avec maladie de von Willebrand est demandée afin de planifier le traitement le plus approprié au moment de l'accouchement.

Dans le type 1 VwD avec FVIII: VWF C inférieur à 30 U/dL, l'administration de la desmopressine généralement après serrage ombilical et pendant 3-4 jours après est nécessaire surtout lorsque l’épisiotomie est nécessaire. La même approche, avec moins de perfusions peuvent être appliquées à ceux qui VWF> 30 et <50 U/dL [5]. Une expérience récente suggère la possibilité de commencer le traitement immédiatement avant l'accouchement, sans effets secondaires évidents pour la mère et le nouveau-né. L'utilisation de la desmopressine au cours du premier trimestre de la grossesse pour couvrir prélèvement de villosités choriales ou amniocentèse semble être faisable et sûre, sans risque de fausse-couche [2].

Dans le type 3 VWD, VWF et de FVIII n'augmentent pas pendant la grossesse et donc les concentrés VWF/FVIII peuvent être nécessaires pendant la grossesse pour contrôler le saignement vaginal intermittent et à **l'accouchement** ou pour une **césarienne**. Cette dernière devrait être réservée seulement pour les indications obstétricales habituelles. Des doses quotidiennes de 50 UI / kg VWF sont tenues de maintenir FVIII: niveau C> 50 U/dL pendant 3-4 jours. Traitement habituel de thromboprophylaxie par HBPM doit être mise en œuvre chez les patients à haut risque de thrombose veineuse au cours du traitement de remplacement en cas de césarienne. Si exécuté, VWF: RCo et FVIII: C de pointe devraient être> 50 U / dL [2].

Chez les patients de type 2A, 2B et 2M généralement VWF: RCo n'atteint pas des niveaux normaux et donc la thérapie de remplacement est de même conseillée. Toutefois, dans le type 2B l'augmentation du VWF anormale peut causer ou aggraver une thrombocytopénie et une transfusion plaquettaire pourraient être également tenue à la parturition [2]. FVIII et VWF chute aux niveaux de base, peu après la livraison, et donc anti-fibrinolytiques oraux (par exemple, l'acide tranexamique 1 gr toutes les 8 heures pendant 3-4 jours) peuvent être utilisés au cours de cette période pour prévenir le saignement du à lochies lourd. Une revue systématique de la littérature récente de 507 techniques d'anesthésie régionale pour des patientes en obstétrique et en dehors de l'obstétrique avec hémophilie, maladie de Von Willebrand ou ITP de 30 articles signale une seule complication dans laquelle la diathèse hémorragique a été non diagnostiquée avant l'insertion de l'aiguille [6]. Il s'agissait d'un cas d'hématome rachidien après ponction lombaire chez une hémophile non conduisant à la paraplégie permanente [6].

## Conclusion

La VWD est la maladie hémorragique constitutionnelle la plus fréquente. Très hétérogène, son diagnostic peut parfois être difficile et demander des tests très spécialisés. La caractérisation du type et du sous type de la maladie est indispensable afin de guider le traitement des patientes mais aussi afin d'adapter notre prise en charge pendant la grossesse. En effet, l’évolution de la pathologie pendant la grossesse et le post-partum varie selon le type de VWD. Les femmes présentant un type 1 voient leur taux de facteurs se normaliser pendant la grossesse. Pour les types 2, l’évolution est variable tandis que pour les types 3, aucune évolution n'est observée.
